# Stride-to-stride fluctuations in transtibial amputees are not affected by changes in push-off mechanics from using different prostheses

**DOI:** 10.1371/journal.pone.0205098

**Published:** 2018-10-03

**Authors:** Chase G. Rock, Shane R. Wurdeman, Nicholas Stergiou, Kota Z. Takahashi

**Affiliations:** 1 Department of Biomechanics, University of Nebraska at Omaha, Omaha, NE, United States of America; 2 Department of Clinical and Scientific Affairs, Hanger Clinic, Houston, TX, United States of America; 3 Department of Environmental, Agricultural, and Occupational Health, University of Nebraska Medical Center, Omaha, NE, United States of America; University of Illinois at Urbana-Champaign, UNITED STATES

## Abstract

Stride-to-stride fluctuations of joint kinematics during walking reflect a highly structured organization that is characteristic of healthy gait. The organization of stride-to-stride fluctuations is disturbed in lower-limb prosthesis users, yet the factors contributing to this difference are unclear. One potential contributor to the changes in stride-to-stride fluctuations is the altered push-off mechanics experienced by passive prosthesis users. The purpose of our study was to determine if changes in push-off mechanics affect stride-to-stride fluctuations in transtibial amputees. Twenty-two unilateral transtibial amputees were enrolled in the 6-week cross-over study, where High and Low Activity (based on the Medicare Functional Classification System) prostheses were worn for three weeks each. Data collection took place at the end of the third week. Participants walked on a treadmill in a motion capture laboratory to quantify stride-to-stride fluctuations of the lower extremity joint angle trajectories using the largest Lyapunov Exponent, and over floor-embedded force platforms to enable calculating push-off work from the prosthesis and the sound limb. Push-off work was 140% greater in the High Activity prosthesis compared to the Low Activity prosthesis (*p* < 0.001), however no significant change was observed in stride-to-stride fluctuations of the ankle between the two prosthesis types (*p* = 0.576). There was no significant correlation between changes in prosthesis push-off work and the largest Lyapunov exponent. Though differences in push-off work were observed between the two prosthesis types, stride-to-stride fluctuations remained similar, indicating that prosthesis propulsion mechanics may not be a strong determinant of stride-to-stride fluctuations in unpowered transtibial prosthesis users.

## Background

During human walking, subtle fluctuations occur from stride to stride, causing the walking pattern to vary over time [1–3]. These stride-to-stride fluctuations are the end behavior of a complex motor system, which includes the various neural, muscular, and mechanical interactions of the body [4]. Such interacting subsystems produce stride-to-stride fluctuations that have a highly structured organization [5–7]. It has been speculated that the temporal organization of the stride-to-stride fluctuations in healthy populations exhibit an optimal state of movement variability which is indicative of high adaptability [3]. Optimal movement variability is observed when a system is neither too restrained nor too disordered [3]. In contrast, stride-to-stride fluctuations exhibit an altered organization in those with compromised neuromuscular systems [8,9]. For example, altered stride-to-stride fluctuations are observed in people who have experienced lower limb loss resulting in reliance on a prosthetic limb for locomotion [10].

When compared to those without amputation, transtibial prosthesis users experience higher stride-to-stride fluctuations [10]; that is, each stride is highly dissimilar to other strides during walking, which signifies a movement pattern that exhibits more divergence between gait cycles. For transtibial prosthesis users, the highest rate of divergence is observed at the prosthetic ankle [10]. Furthermore, prosthesis users may perceive changes in stride-to-stride fluctuations at the prosthetic ankle. For example, when given a choice between two different prostheses, individuals tend to prefer a prosthesis that results in lower levels of stride-to-stride fluctuations, (i.e. a less divergent ankle trajectory) [11]. In other words, they prefer a prosthesis that more closely resembles the stride-to-stride fluctuations observed in those without amputation. Despite these experimental findings, the specific source of the differing stride-to-stride fluctuations in prosthesis users remains unclear. The stride-to-stride fluctuations may be influenced by various factors, including the mechanical properties of the prosthesis, neuromuscular adaptation and control, or sensory feedback at the interface with the prosthesis.

We propose that the altered stride-to-stride fluctuations in lower-limb prosthesis users may be, in part, due to impaired propulsion mechanics. Propulsion is an essential feature of locomotion and can be severely affected by lower-limb amputation [12–14]. Both modeling and empirical observations have provided support for a potential link between stride-to-stride fluctuations and propulsion mechanics. For example, in a dynamic walking model, increased toe-off impulse during propulsion induced increased stride-to-stride fluctuations [15]. Similarly, an experiment in human walking has revealed that increased assistive propulsive force leads to increased stride-to-stride fluctuations [15]. One important characteristic of passive lower limb prostheses is their inability to generate different levels of propulsion on their own [16,17]; unpowered prostheses can only store and return the energy they receive (similar to a spring), rather than actively produce force and work (similar to a motor or muscle) [18]. The spring-like mechanism used by unpowered prostheses results in power delivery during late stance (i.e. push-off work) that typically does not equal the push-off work of a biological limb [12,14,16,17,19,20].

It is interesting to note that both *increased* propulsion beyond typical levels (as in studies using assistive propulsion [15]) and *decreased* propulsion (as in prosthesis users [12]) lead to increased stride-to-stride fluctuations when compared to normal, healthy adults. Thus, the effects of propulsion on stride-to-stride fluctuations may not be a linear relationship, but rather a non-linear trend where an ‘optimal’ level of push-off (i.e., comparable to levels of healthy individuals) results in reduced stride-to-stride fluctuations. Therefore, it may be that the reduced push-off work from the prosthesis could influence increases in stride-to-stride fluctuations in prosthesis users.

Observations from other clinical populations may shed light on the potential link between push-off mechanics and stride-to-stride fluctuations. In the elderly, sarcopenia and other factors contribute to reduced levels of propulsion [21–23] when compared to younger adults. The elderly also experience increased stride-to-stride fluctuations at the hip, knee, and ankle [8]. In persons with peripheral arterial disease, progressive loss of muscular tissue hinders the strength of the plantarflexors [24], likely inhibiting their ability to generate push-off work [25]. In addition, these patients exhibit increased stride-to-stride fluctuations when compared with age-matched controls [9]. From these observations in the elderly and in those with vascular disease, it would seem that a diminished ability to generate push-off work coincides with increased levels of stride-to-stride fluctuations. While prosthesis users and pathological populations experience comorbidities that may affect movement (e.g., neuropathy [26]), these are difficult to isolate. However, altering the prosthesis mechanics represents a unique opportunity to directly investigate how changes in push-off work may be linked to stride-to-stride fluctuations.

The purpose of this study was to determine the relationship between prosthesis push-off work and stride-to-stride fluctuations. In order to explore this research question, data were reanalyzed from Wurdeman et al., 2014 [27]. In this study, prosthesis users wore two different prosthesis types for 3 weeks each: a ‘High Activity’ and a ‘Low Activity’ prosthesis, according to the Medicare Functional Classification System. The two types of prostheses were expected to produce different energy storage-and-return characteristics (higher energy return from the High Activity prosthesis), in accord with previous research comparing High and Low Activity prostheses [28,29]. It was shown previously that 3 weeks of adaptation can significantly influence stride-to-stride fluctuations [27]. In order to mitigate the effect of such adaptation, we reanalyzed data from only the third week; thus enabling us to examine the isolated effect of propulsion mechanics on stride-to-stride fluctuations. We hypothesized that 1) ankle-foot push-off work would be greater from the High Activity prostheses, 2) stride-to-stride fluctuations would be reduced in the High Activity prostheses, and 3) that the difference in ankle-foot push-off work provided by the two types of prostheses would be correlated with the difference in stride-to-stride fluctuations between the prostheses.

## Materials and methods

### Participants

The data from 22 participants (nine with right leg amputation and thirteen with left leg amputation; Table 1) were reanalyzed from the original Wurdeman et al. 2014 dataset [27]. Participants were included in this analysis if they were unilateral, transtibial prosthesis users who completed the 6-week protocol detailed in the Wurdeman et al. 2014 publication [27]. All participants were classified as K-level 3 or 4 according to the Medicare Functional Classification System.

**Table 1:** Participant demographic information.

Age (yrs)	Time Since Amputation (yrs)	Height (m)	Mass (kg)	Cause of Amputation
53.0 ± 11.8	7.5 ± 6.0	1.77 ± 8.23	101.5 ± 18.7	Trauma—13 Vascular/diabetes– 6 Infection– 2 Cancer—1

Mean ± Standard Deviation

### Ethics statement

All procedures were approved by the University of Nebraska Medical Center and the Nebraska/Western Iowa Veterans Affairs Medical Center Institutional Review Boards.

### Experimental procedure

All details about the 6-week randomized cross-over study can be found in the original report by Wurdeman et. al. The data presented in this study are from the final data collection, after the participants had been wearing each prosthesis for three weeks. In brief, each participant was first fitted with a new prosthetic foot that either matched the activity level (K3 or K4, Medicare Functional Classification System) of their current foot or was rated for lower activity (K2, Medicare Functional Classification System) than their current foot. The participant then wore the new prosthetic foot during their daily activities for the next three weeks, in order to acclimatize them to the mechanical properties of the new prosthesis. At the end of this period a gait analysis was performed and then the participant was fitted with a second prosthetic foot (High Activity or Low Activity) depending on which foot they had received for the previous 3 weeks. They again wore this new prosthetic foot for the next three weeks, after which gait analysis was performed. High Activity prostheses included devices made by (numbers indicate number of participants assigned each foot): Ability Dynamics (Rush Foot– 3), Endolite (Elite– 1), Freedom Innovations (Pacifica– 2, Renegade– 4, Renegade Torsion– 1, Senator– 3), Ossur (Variflex– 2), and Willowwood (Duralite– 2, Fusion– 4). For Low Activity prostheses, 21 participants wore a SACH foot, while one participant wore a Walktek by Freedom Innovations.

#### Data capture

**Fig 1:**
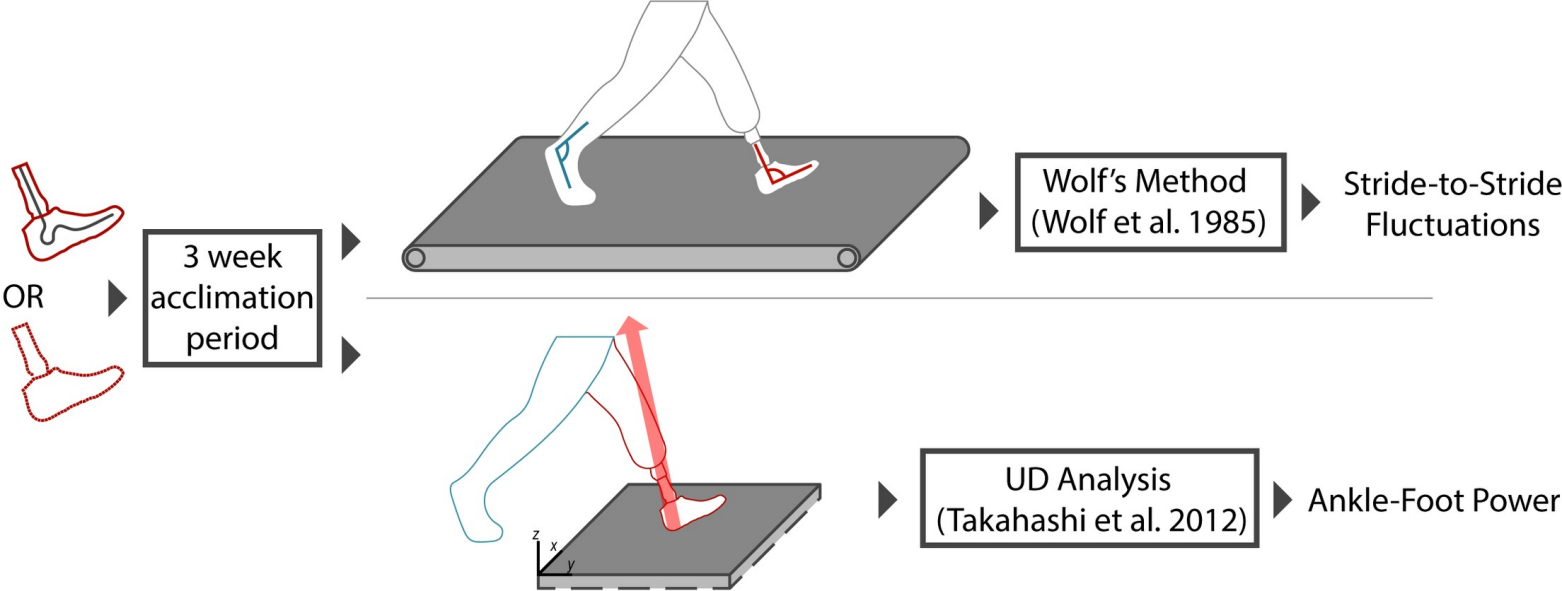
Participants were first fitted with a new prosthetic foot that was classified as either High Activity (HA) or Low Activity (LA) according to the Medicare Functional Classification System. Participants then wore the new prosthetic foot for a period of three weeks to acclimate to the new device. After the three-week period, participants underwent gait analysis during both treadmill and overground walking. Treadmill walking consisted of three minutes at subject-selected speed. Continuous ankle angle data from a single treadmill walking bout (obtained using infrared cameras in conjunction with retroreflective markers on the shoes, prosthesis, and legs) was used to calculate stride-to-stride fluctuations via Lyapunov exponent for both limbs. Overground trials were completed at self-selected walking speed, and force data were recorded using a Kistler force platform to calculate ankle-foot power.

Gait analysis was performed at the end of each three-week acclimation period. Twenty-seven retroreflective markers were attached to various anatomical landmarks on the lower body. On the prosthetic limb, markers were placed in locations analogous to the sound limb. The three-dimensional data from these markers were captured by a 12-camera motion analysis system (Motion Analysis Corp, Santa Rosa, CA, USA; sampled at 60 Hz) which allowed for the measurement of lower limb segment motion. Subjects performed 1) treadmill walking at their preferred walking speed lasting 3 minutes and 2) overground walking at their self-selected speed over a floor-embedded force-plate (Fig 1). Treadmill walking was performed at a subject-selected speed, which was maintained for both the High and Low Activity conditions (average speed = 0.89 ± 0.38 m/s). At least five overground trials per foot (10 total trials) were captured for each subject where the foot was in clear contact with the force-plate [30].

#### Data analysis

The ankle, knee, and hip flexion/extension angles were calculated (Visual3D, Germantown, MD, USA) for the sound and prosthetic limbs from the treadmill walking data. The largest Lyapunov exponent [31–33] was then calculated for each limb using the continuous ankle, knee, or hip angle data. The largest Lyapunov exponent is a measure of the rate of divergence across many steps [10]. Low divergence (lower Lyapunov exponent) indicates that each step is similar to the steps that precede/follow it. In contrast, high divergence (higher Lyapunov exponent) denotes dissimilarity across steps.

Briefly, the process for calculating Lyapunov exponent begins with plotting the ankle angle data against a time-delayed version of itself. This results in a signal that has higher dimensionality than the original signal allowing for more information to be gained. The proper dimension is determined using the false nearest neighbor algorithm and the proper time lag is determined using the average mutual information algorithm [27]. From this reconstructed signal, a point is selected and compared to a nearby point on a different trajectory. This pair of points is followed for a certain number of iterations, where the distance between them is measured [31]. From this, the rate at which the points diverge or converge (i.e. Lyapunov exponent) can be calculated. This process is repeated along the entire reconstructed signal, at every dimension. As such, a Lyapunov exponent is calculated for every dimension. The largest of these exponents, namely the largest Lyapunov exponent which describes the dynamics of the system as a whole was used in this study.

From the overground trials, ankle-foot power was calculated for the prosthetic limb and for the intact ankle-foot using unified deformable (UD) segment analysis [34]. This analysis captures the summed contribution of all structures distal to the shank (i.e., anatomical ankle-foot, or prosthesis). The UD analysis does not require defining of an ankle joint, facilitating direct comparisons between various prosthetic types and anatomical limbs. The calculation for UD segment power requires four input variables: Ground reaction force (${\bar{F}}_{GRF}$), the free moment (${\bar{M}}_{free}$), angular velocity of the shank ($\bar{\omega }$), and velocity of distal structures (${\bar{v}}_{d}$), which is obtained using the equation:

$${\bar{v}}_{d}={\bar{v}}_{cm}+\left(\bar{\omega }\times {\bar{r}}_{COP}\right)$$ (1)

where ${\bar{v}}_{cm}$ is the translational velocity of the center of mass of the shank, and ${\bar{r}}_{COP}$ is the vector from the shank center of mass to the COP. Once ${\bar{v}}_{d}$ is known, we can use the following equation to quantify the total power absorbed, dissipated, returned or generation by all structures distal to the shank:

$$P_{UD}={\bar{F}}_{GRF}\cdot {\bar{v}}_{d}+{\bar{M}}_{free}\cdot \bar{\omega }$$ (2)

**Fig 2:**
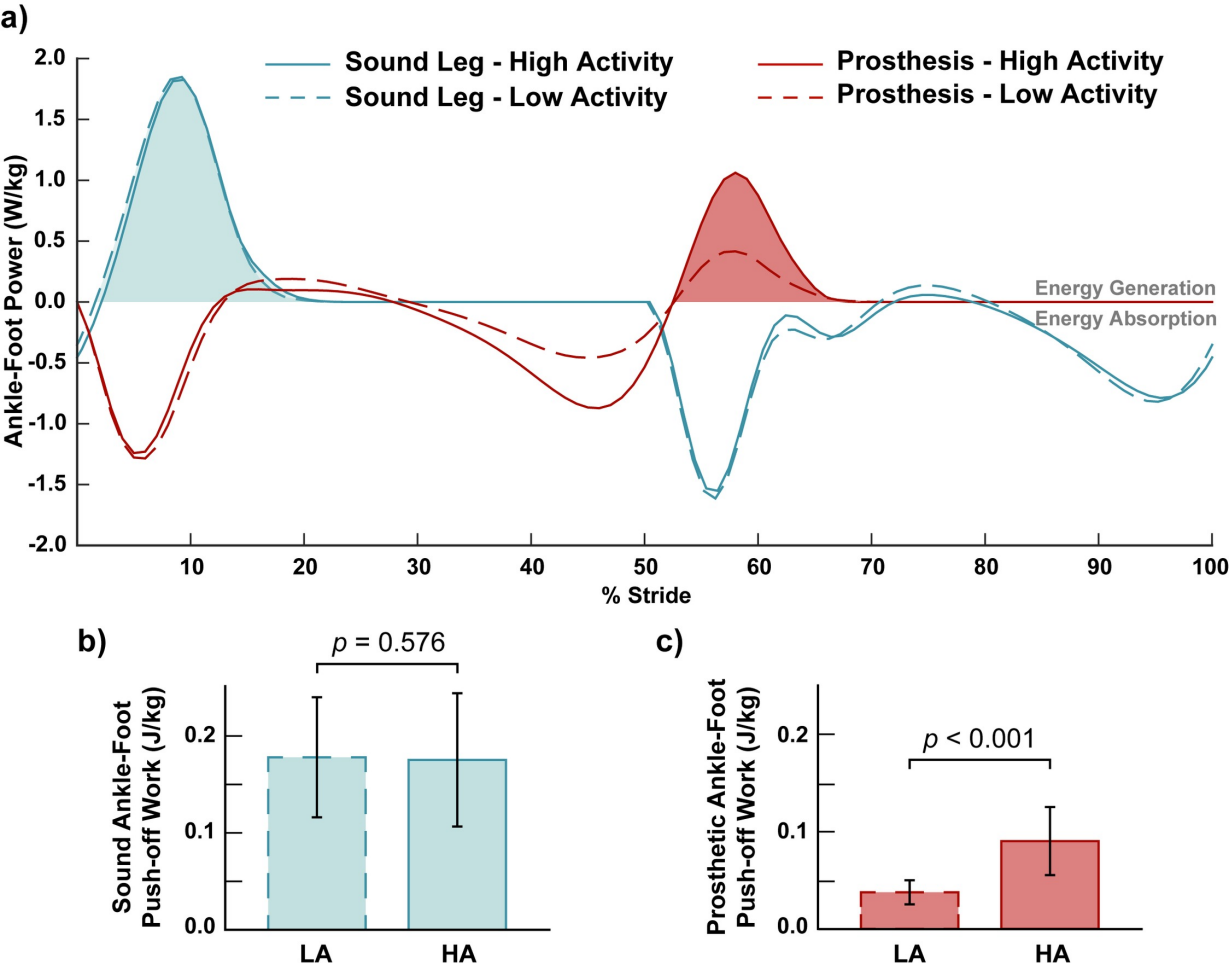
Prosthesis push-off work was 140% greater in the High Activity (HA) prostheses compared to the Low Activity (LA) prostheses (p < 0.001). Sound limb push-off work showed no significant difference between the two prosthesis conditions (p = 0.576). The late-stance positive power was integrated (a, shaded regions) to determine push-off work from the sound (b) and prosthetic (c) ankle-foot structures during overground walking. This process was completed for each of four limb conditions: sound limb, contralateral to low activity prosthesis (blue, dashed line); sound limb, contralateral to high activity prosthesis (blue, solid line); prosthetic limb, low activity prosthesis (red, dashed line): prosthetic limb, high activity prosthesis (red, solid line).

To quantify mechanical work, power (*P*_*UD*_) was integrated with respect to time. Specifically, to quantify the work done during late stance (i.e. push-off), the portion of positive power during late stance was integrated (Fig 2A, shaded regions). For the prosthesis, this provides a measure of the energy returned from the prosthesis during late stance. For the sound limb, this measures the overall energy output (generated or returned) from the foot and ankle structures during push-off.

#### Statistical analysis

A paired t-test was used to identify if the largest Lyapunov exponent was different between the High Activity or Low Activity prosthetic foot. A paired t-test was also used to identify differences in push-off work (for both the intact and the prosthetic ankle-foot) between the High Activity and Low Activity trials. The change in largest Lyapunov exponent and prosthetic push-off work was calculated by subtracting the value calculated for the Low Activity trials from the High Activity values. A Pearson’s correlation was used to determine how changes in prosthesis push-off work correlated with changes in the largest Lyapunov exponent of the prosthetic and sound ankle.

## Results

### Push-off work between prostheses

The mechanical power profiles from both High Activity and Low Activity prosthetic ankle-foot during walking were characterized by negative work during early stance, indicative of energy storage and/or dissipation (Fig 2A). During late stance, some of this stored energy was returned in the form of positive work (i.e. push-off work). Push-off work from the High Activity prosthesis (0.090 ± 0.034 J/kg; mean ± standard deviation) was 140% greater than that from the Low Activity prosthesis (0.038 ± 0.012 J/kg; *p* < 0.001: Fig 2C). In contrast, the push-off work from the intact limb (Fig 2B) was not significantly different (*p* = 0.576) in the High Activity condition (0.175 ± 0.069 J/kg) when compared to the Low Activity condition (0.178 ± 0.062 J/kg). Overground walking speed was not significantly different between the High (1.25 ± 0.25 m/s) and Low Activity (1.26 ± 0.25 m/s) prostheses (*p* = 0.323).

### Stride-to-stride fluctuations between prostheses

**Fig 3:**
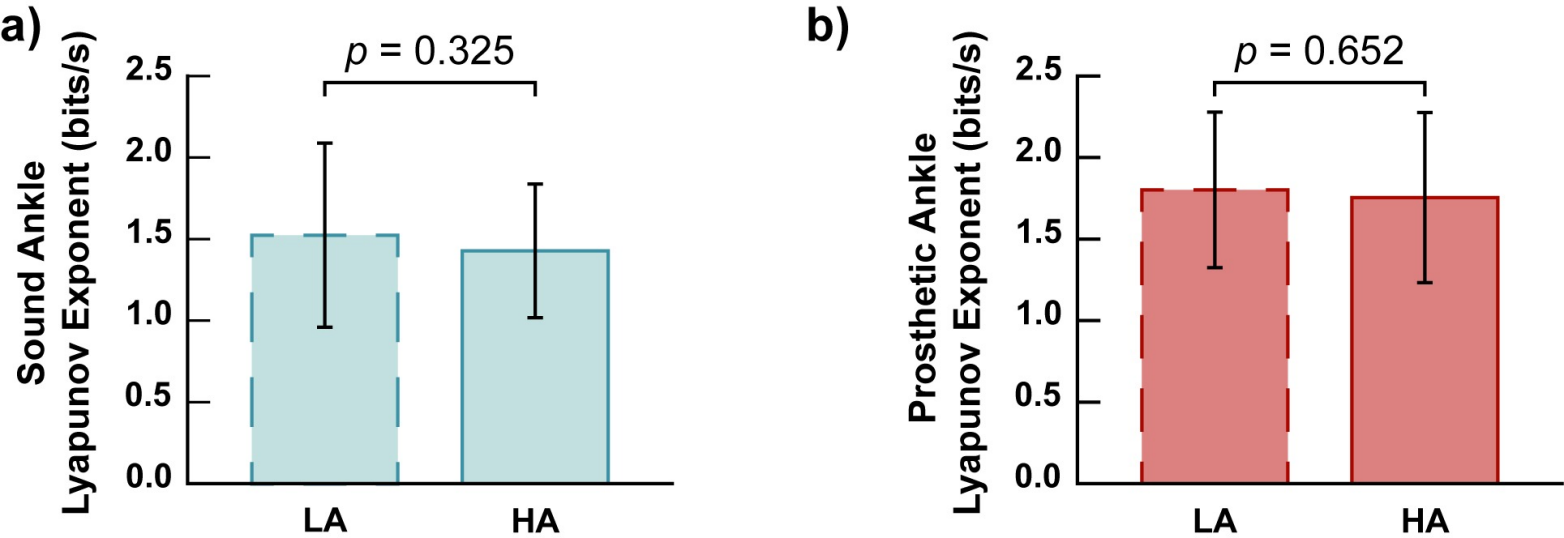
Stride-to-stride fluctuations at the ankle were not significantly different between high and low activity prostheses for the sound limb (a, p = 0.325) or the prosthetic limb (b, p = 0.652). Lyapunov exponent is a method of characterizing the fluctuations of a repetitive action. Larger values of Lyapunov exponent indicate increased divergence in the gait pattern while smaller values indicate lower divergence.

The largest Lyapunov exponent (Fig 3) of the prosthetic ankle was not significantly different (*p* = 0.652) between the High Activity (1.75 ± 0.52 bit/s) and Low Activity prostheses (1.80 ± 0.48 bit/s). At the intact ankle, no significant difference (*p* = 0.325) was observed in largest Lyapunov exponent between the High Activity (1.43 ± 0.41 bit/s) and Low Activity (1.53 ± 0.57 bit/s) conditions.

As an additional analysis, the stride-to-stride fluctuations of the hip and knee were compared between prostheses. The largest Lyapunov exponent at the hip and knee were not significantly different (*p* = 0.393 and *p* = 0.736, respectively) between the High Activity (hip: 0.66 ± 0.14 bit/s; knee: 1.02 ± 0.37 bit/s) and Low Activity Prostheses (hip: 0.63 ± 0.17 bit/s; knee: 1.00 ± 0.27 bit/s) on the amputated leg. For the intact limb, no significant difference was observed at the hip (*p* = 0.317) or knee (*p* = 0.274) between the High Activity (hip: 0.64 ± 0.17 bit/s; knee: 0.98± 0.27 bit/s) and Low Activity (hip: 0.60 ± 0.11 bit/s; knee: 1.05 ± 0.35 bit/s) prostheses. Subject-specific values for stride-to-stride fluctuations and push-off work are available as Supporting Information (S1 Table).

### Relationship between push-off work and stride-to-stride fluctuations

**Fig 4:**
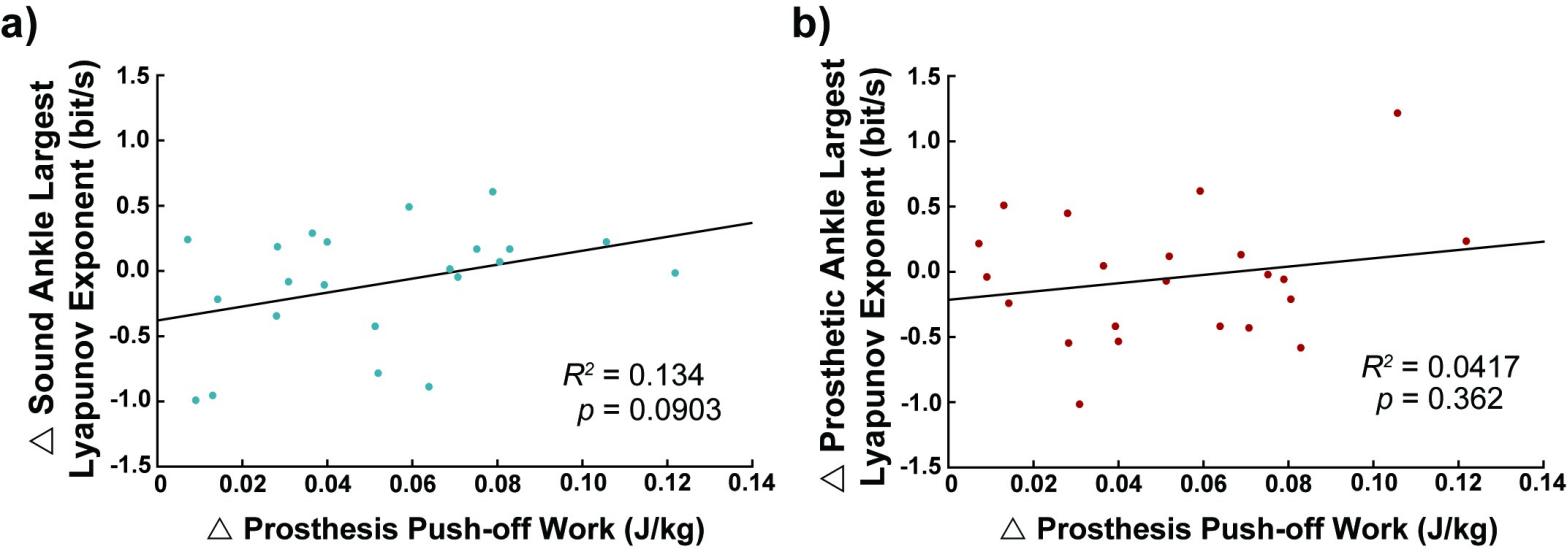
**Changes in prosthesis push-off work did not correlate with stride-to-stride fluctuation changes within individuals at the sound ankle (a, R^2^ = 0.134, n = 22, p = 0.0903) or the prosthetic ankle (b, R^2^ = 0.0417, n = 22, p = 0.362).** As stride-to-stride fluctuations result from the interaction of various systems including neural, mechanical, and (in this case) synthetic, we expected that changes to the prosthesis mechanics would result in changes in the stride-to-stride fluctuations. The lack of relation indicates that the mechanical contribution of the limb to stride-to-stride fluctuations may not be a significant contributor. (Δ = High Activity prosthesis value–Low Activity prosthesis value).

No correlation was observed between change in prosthesis push-off work and change in stride-to-stride fluctuations at the sound ankle (*R*^*2*^ = 0.134, *p* = 0.090; Fig 4A) or the prosthetic (*R*^*2*^ = 0.042, *p* = 0.362; Fig 4B) ankle.

Additionally, no significant correlation was observed between changes in prosthesis push-off work and change in stride-to-stride fluctuations at the prosthesis side knee (*R*^*2*^ = 0.080, *p* = 0.203) or hip (*R*^*2*^ = 0.100, *p* = 0.153), nor at the intact side knee (*R*^*2*^ = 0.006, *p* = 0.733) or hip (*R*^*2*^ = 0.003, *p* = 0.822).

## Discussion

Our aim was to investigate how prostheses with different energy return characteristics affect stride-to-stride fluctuations at the ankle in individuals with a unilateral transtibial amputation. By calculating the push-off work of Low Activity and High Activity prostheses in the same user, we were able to specify how the prosthesis types differed in function. In addition, we investigated the stride-to-stride fluctuations of the sound and prosthetic ankle angle, in order to detect how stride-to-stride fluctuations may be affected by different prostheses.

While both types of prostheses are able to store and return energy, the High Activity prostheses are specifically designed to return a higher amount of energy than the Low Activity prostheses. The push-off work measured from the prosthesis was approximately 140% higher in the High Activity prostheses (0.090 J/kg) when compared to the Low Activity prostheses (0.038 J/kg), which was consistent with our hypothesis. This change in work is similar in magnitude to a person without amputation increasing walking speed by approximately 0.34 m/s [35]. However, no change in walking speed was observed in the current study, indicating that not all of the increased push-off work was translated into energy for forward propulsion, perhaps due to changes in knee or hip power, or energy that was dissipated in the prosthesis-residual limb interface. Nonetheless, the push-off work from the High Activity prostheses came closer to resembling the sound limb when compared to the Low Activity prostheses. The sound limb produced push-off work that was less than double the value from the High Activity prostheses. In comparison, the sound limb push-off work was more than quadruple the Low Activity push-off work. These differences are consistent with previous measures of total positive work between a High Activity prosthesis and the sound limb, where the sound limb produced slightly more than double the work of the prosthesis [19].

Based on prior research that identified a link between propulsion mechanics and stride-to-stride fluctuations [15], we hypothesized that changes in push-off work between prostheses would lead to changes in stride-to-stride fluctuations. However, contrary to our hypothesis, no change was observed in stride-to-stride fluctuations. One potential reason for the lack of change in stride-to-stride fluctuations could be that the difference in push-off work between the two prostheses was not large enough. Even though the push-off work in the High Activity prosthesis was more than double that of the Low Activity prosthesis, the absolute magnitude of this difference was about 0.05 J/kg, which equates to about 29% of the push-off work from the sound limb. In future work, a greater range of prosthesis push-off work may be accomplished by the use of powered prostheses [17,19,36] that can systematically modulate the work output. It should also be noted that the overground and treadmill trials had different speeds (approximately 1.25 and 0.89 m/s, respectively). Though speed can affect both push-off work [35] and stride-to-stride fluctuations [37], the main comparison in this study was between prosthesis types, and subjects walked at the same speeds with both prosthesis types. Lastly, mechanical differences other than push-off work between the High and Low Activity prostheses may have affected stride-to-stride fluctuations. Differences in factors such as joint articulation, shape, and cushioning may have caused subtle changes in stride-to-stride fluctuations, offsetting the potential contribution of push-off work. Future efforts should investigate the specific contributions of these mechanical factors to stride-to-stride fluctuations.

In contrast to the results from the current study, a previous study found a significant effect of propulsion mechanics on stride-to-stride fluctuations using horizontal forces of up to 9% body weight [15]. These additional forces were supplied by pulling on a belt attached to the waist, a method that is different from our method in a few ways. First, waist-belt assistive forces do not have an everyday analogue and therefore may be perceived as a disturbance. Second, participants have no direct level of control over waist-belt forces, whereas the forces received by a passive prosthesis can be controlled by changing the way it is loaded. Lastly, waist-belt forces likely caused a larger difference in propulsion than the effect observed in the current study. Also in the previous study, anterior/posterior forces were manipulated while stride-to-stride fluctuations were simultaneously recorded [15].

The current study measured push-off work and stride-to-stride fluctuations from separate overground and treadmill trials, making direct comparisons difficult. Though propulsion has been shown to be comparable between overground and treadmill walking [38], it is difficult to determine if stride-to-stride fluctuations during treadmill walking are directly transferrable to overground walking. In order to address some of these issues, future studies need to examine a larger range of push-off work from the prostheses and should simultaneously measure push-off work and stride-to-stride fluctuations, perhaps via an instrumented treadmill.

Previous research has shown how the stride-to-stride fluctuations of prosthesis users differ from those without amputation [10]. Because the manipulation of push-off work employed in the current study did not elicit changes in stride-to-stride fluctuations, it may be that the determinants of stride-to-stride fluctuations in unpowered prosthesis users are non-mechanical. Stride-to-stride fluctuations are the end behavior of the human neuromuscular system interacting with the environment, and thus inter-stride variability in prosthesis users could be affected by several factors other than prosthetic push-off mechanics. For example, a prior study by Wurdeman et al showed that adaptation of the neuromuscular system to the prosthesis can affect stride-to-stride fluctuations [27]. This current study reanalyzed a subset of the data analyzed by Wurdeman et. al [27] (i.e., data only from the third week of adaptation), to mitigate the effect of the adaptation process. The present findings reflect the trends observed by Wurdeman et. al [27], in that stride-to-stride fluctuations appear to be highest at the ankle and lowest at the hip. Furthermore, because we found no difference in stride-to-stride fluctuations between High Activity and Low Activity prostheses after the adaptation period, it may be that stride-to-stride fluctuations are more influenced by adaptation to the prosthesis than the mechanical push-off work.

Additionally, factors other than push-off work, such as altered proprioception and muscle weakness, could affect stride-to-stride fluctuations. For example, in older adults and people with peripheral arterial disease, altered stride-to-stride fluctuations [8,9] are accompanied by a reduction in push-off work [21–25]. However, these populations also have systemic deficits in sensation, perhaps due to neuropathy [26], as well as muscle weakness [21]. Considering these deficits in conjunction with the results from the current study, we speculate that sensation (e.g., proprioception) may be a key determinant of stride-to-stride fluctuations. Indeed, sensory feedback is diminished in prosthesis users due to the nature of amputation (severed nerve and muscle) as well as the less-than-ideal interface between the residual limb and socket. Future projects could explore how augmenting sensory feedback from the prosthetic limb may affect inter-stride variability, possibly by augmenting the sensory feedback of the residual limb [39], or by artificially substituting sensory feedback signals [40–42].

## Conclusion

We aimed to study how the changes in push-off work from prostheses may affect changes in stride-to-stride fluctuations. Distinctly different push-off work was observed between High and Low Activity prostheses. Push-off work from the High Activity prosthesis equaled about 50% of the push-off work value from the sound limb, while the Low Activity prosthesis provided less than 25%. Despite these differences in push-off work between the prostheses in this study, there was no significant change in the stride-to-stride fluctuations of the ankle, knee, and hip flexion angles. Stride-to-stride fluctuations may not be sensitive to the difference in prosthesis push-off work achieved by different prostheses (i.e., High Activity vs Low Activity), implying that stride-to-stride fluctuations arise from other, perhaps non-mechanical, factors.

## Supporting information

S1 Table Subject-specific values for stride-to-stride fluctuations (bit/s) and push-off work (J/kg). Individual values for each leg (prosthesis side or sound side) and each condition (High Activity or Low Activity prosthesis) are reported. For stride-to-stride fluctuations, values were calculated at the ankle, knee, and hip.
(XLSX)
